# Recognition of Double Strand Breaks by a Mutator Protein (MU2) in *Drosophila melanogaster*

**DOI:** 10.1371/journal.pgen.1000473

**Published:** 2009-05-08

**Authors:** Raghuvar Dronamraju, James M. Mason

**Affiliations:** Laboratory of Molecular Genetics, National Institute of Environmental Health Sciences, Research Triangle Park, North Carolina, United States of America; Stowers Institute for Medical Research, United States of America

## Abstract

Telomere capture, a rare event that stabilizes chromosome breaks, is associated with certain genetic abnormalities in humans. Studies pertaining to the generation, maintenance, and biological effects of telomere formation are limited in metazoans. A mutation, *mu2^a^*, in *Drosophila melanogaster* decreases the rate of repair of double strand DNA breaks in oocytes, thus leading to chromosomes that have lost a natural telomere and gained a new telomere. Amino acid sequence, domain architecture, and protein interactions suggest that MU2 is an ortholog of human MDC1. The MU2 protein is a component of meiotic recombination foci and localizes to repair foci in S2 cells after irradiation in a manner similar to that of phosphorylated histone variant H2Av. Domain searches indicated that the protein contains an N-terminal FHA domain and a C-terminal tandem BRCT domain. Peptide pull-down studies showed that the BRCT domain interacts with phosphorylated H2Av, while the FHA domain interacts with the complex of MRE11, RAD50, and NBS. A frameshift mutation that eliminates the MU2 BRCT domain decreases the number and size of meiotic phospho-H2Av foci. MU2 is also required for the intra-S checkpoint in eye-antennal imaginal discs. MU2 participates at an early stage in the recognition of DNA damage at a step that is prerequisite for both DNA repair and cell cycle checkpoint control. We propose a model suggesting that neotelomeres may arise when radiation-induced chromosome breaks fail to be repaired, fail to arrest progression through meiosis, and are deposited in the zygote, where cell cycle control is absent and rapid rounds of replication and telomere formation ensue.

## Author Summary

Telomeres are structures at the ends of eukaryotic chromosomes required for chromosome stability. If unrepaired, a single chromosome end without a telomere is sufficient to kill a cell, but new telomere formation is rare. Previously, we described a gene in *Drosophila* whose mutants, after irradiation, produced many progeny with chromosomes lacking a natural telomere. The new broken chromosome ends, however, bound telomeric proteins and behaved as telomeres. Here, we show that the protein encoded by this gene, a homolog of the human *MDC1* gene, is a component of the repair foci that form at double strand DNA breaks and are prerequisite for both cell cycle arrest and DNA repair. The protein acts as a scaffold, connecting a phosphorylated histone that marks the site of the break to a protein complex necessary for repair. These results suggest a model for formation of neotelomeres in which DNA breaks induced in mutant oocytes evade repair and are deposited into embryos, which contain an abundance of maternally deposited telomeric proteins. In this context a chromosome end not recognized as broken may be treated as a telomere. These results may provide a basis to understand neotelomere formation.

## Introduction

A single unrepaired DNA double strand break (DSB) in a dividing cell is a potentially lethal event. DSBs are generated naturally upon the collapse of replication fork [1], genome rearrangement by yeast mating type switching [2], V(D)J recombination [3], meiosis [4],[5] and exogenous damage. Two main pathways implicated in the repair of a DSB are homologous recombination (HR) and nonhomologous end joining (NHEJ). A cell responds to a DSB by recruiting a host of DNA damage response (DDR) proteins to the chromatin sites near the DSB [6]. While most of the DDR proteins function in either HR or NHEJ, a number of them influence both pathways, including the MRE11/RAD50/NBS1 (MRN) complex, BRCA1, histone H2AX, DNA PKcs and ATM [7]–[9]. A high degree of conservation in DSB repair systems makes it convenient to use a model organism such as *Drosophila* to investigate these basic processes. Thus, it may also be possible to obtain and characterize mutations for specific genes necessary for DNA repair.

Unrepaired broken chromosome ends are highly unstable, initiating cell cycle arrest or cycling through repeated breakage-fusion-bridge cycles [10]. In the germline they are rarely transmitted to the next generation [11], although occasionally these broken chromosomes are stabilized and are propagated normally [12]. The mechanism of de novo telomere addition to nontelomeric DNA is unclear, but in systems using telomerase to maintain telomeres the enzyme telomerase is known to play an important role [13]. The bulk of our knowledge concerning de novo telomere formation comes from studying ciliates that undergo developmentally programmed chromosome fragmentation and healing [14]. In most organisms, the only evidence for telomere loss is through the detection of aberrant karyotypes including terminal chromosome deficiencies that are successfully propagated as a result of de novo telomere formation. Terminal deficiencies are frequent in tumors [15], as exemplified by malignant melanomas in humans containing deletions of chromosome 6 [16]. Given the consequences of terminal deficiencies in humans, it would be of interest to study the nature and mechanistic details of their generation and maintenance in a genetically amenable organism such as *Drosophila*.

An ionizing radiation-dependent, female-specific mutator (*mu2^a^*) on chromosome 3 of *Drosophila melanogaster* has been described that specifically increases the recovery of terminal deficiencies, i.e., chromosomes that have lost a natural telomere. Mature *mu2^a^* oocytes when treated with 5 Gy of γ irradiation show an increase in the frequency of *y* mutants over the controls. Cytological and molecular analysis of the *y* mutant chromosomes from *mu2^a^* mothers showed that the great majority were terminal deficiencies [17]. *mu2^a^* females exhibited a delay in the repair of γ ray induced lesions in the oocyte [17], as well as reduced meiotic recombination [18]. Although lesions induced in mature sperm are repaired after fertilization under the genetic control of female to which the males are mated [19], these lesions do not potentiate terminal deficiencies when the female carries the *mu2^a^* mutation, suggesting that the DNA repair machinery, per se, is not affected by this mutation. In addition, the transmission of DSBs from the oocyte to the zygote suggests a defect in cell cycle regulation.

We have endeavored to identify the function of MU2 and understand phenotype of *mu2^a^* at the molecular level. mu2 mRNA encodes a polypeptide of ∼139 kDa [20] that has a tandem BRCA1 C-terminal (BRCT) domain at the C terminus. We show here that the MU2 protein is associated with ionizing radiation-induced foci in somatic cells and recombination foci during meiosis. The N terminus of MU2 contains a forkhead associated (FHA) domain that interacts with the MRN complex, while the BRCT domain interacts with histone variant H2Av phosphorylated at Ser 137 (γH2Av). This histone variant is homologous to mammalian H2AX [21], which when phosporylated marks DNA repair foci. MU2 also plays a role in the regulation of the intra-S checkpoint in eye-antennal imaginal discs. Based on amino acid alignment, functional similarities, domain architecture, and protein-protein associations, we propose that *mu2* is an ortholog of human *mediator of DNA damage checkpoint 1* (*MDC1*) gene. Sequencing of the *mu2^a^* cDNA revealed a frameshift mutation at aa 1065 leading to a stop codon and loss of the BRCT domain. The delay in the repair of radiation induced lesions in conjunction with the loss of cell cycle control in *mu2^a^* mutants suggests a novel role of the mutation in the formation of new telomeres at the site of a DSB.

## Results

### Location of MU2 In Vivo

**Figure 1:**
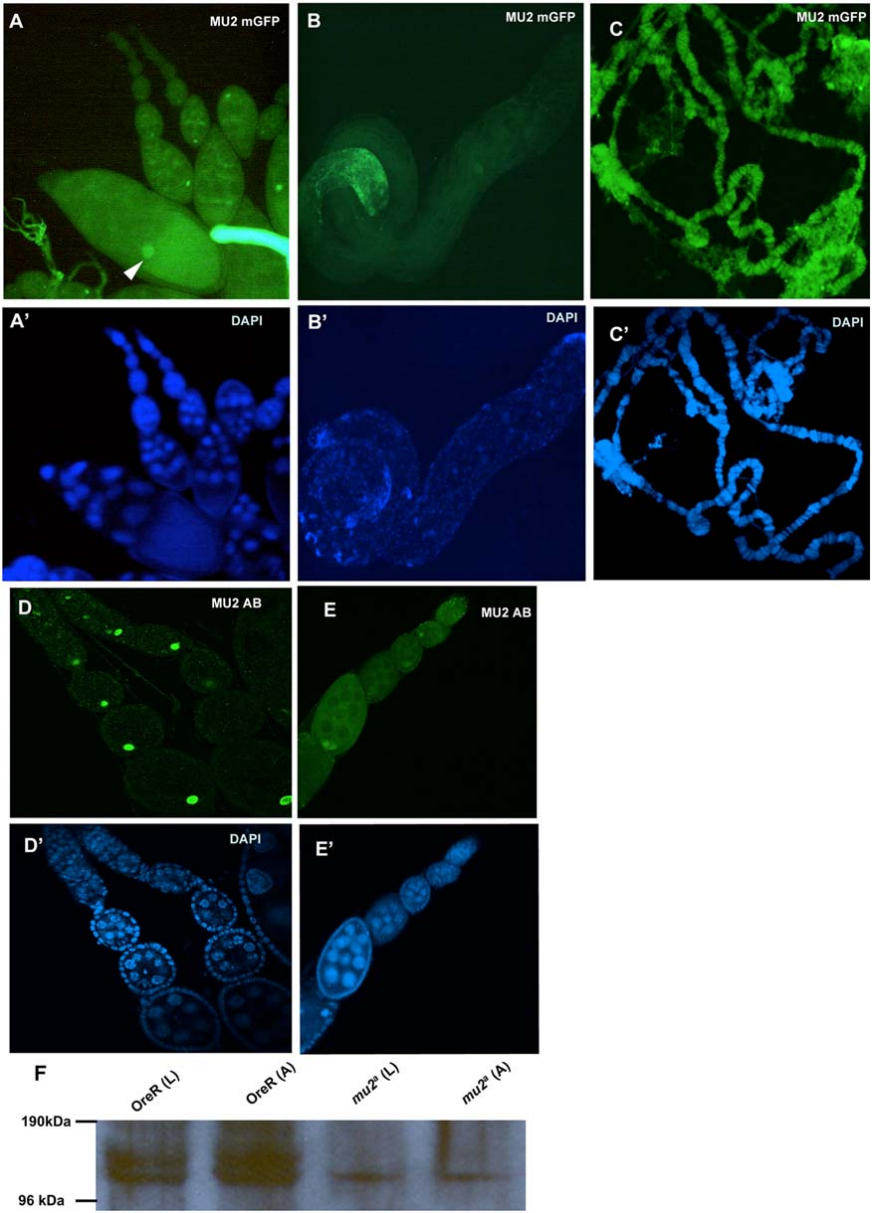
Localization of MU2 protein. (A) Ovaries transgenic for mGFP-tagged MU2 were examined for mGFP fluorescence (green) and (A′) counterstained with DAPI. The oocyte nucleus and cytoplasm were fluorescent although the nucleus is brighter (arrowhead). (B) Testes from the transgenic animals were examined for mGFP fluorescence and (B′) DAPI staining. Fluorescence is not visible in the male germ cells, but is visible in somatic sheath cells. (C) Polytene chromosomes from salivary glands of transgenic animals carrying mGFP-tagged MU2 were examined for fluorescence. The localization of mGFP-MU2 was similar to the DAPI staining (C′). (D) Immmunostaining of Oregon R ovaries using anti-MU2 antiserum showed that MU2 is present primarily in the oocyte nucleus. The fluorescent signal in the oocyte nucleus may be more pronounced than in panel A. (E) *mu2^a^* oocytes immunostained with anti-MU2 antibody and (E′) with DAPI. The overall fluorescence intensity is much less than in panel D. (F) Mutant (*mu2^a^*) and wild type (OreR) larval (L) and the adult (A) extracts were prepared using RIPA buffer, and the MU2 protein in the extracts was detected using mouse anti-MU2 antiserum.

Genetic studies suggested that oocytes mutant for *mu2* have a defect in the processing of DSBs induced by ionizing radiation and produce terminally deficient chromosomes at a high frequency [12],[17]. Based on phenotypic differences between males and females, it was proposed that the MU2 protein is a component of the oocyte nucleus. To understand the localization of MU2, transgenic animals expressing modified GFP (mGFP) tagged MU2 were made. Oocytes from these females showed that MU2 is concentrated in the oocyte nucleus at several stages of development (Figure 1A), although the cytoplasm of more advanced oocytes was also fluorescent. The testes of transgenic males do not show any MU2 localization to the germ cells (Figure 1B), although tagged MU2 localized to the somatic sheath cells, as was found for mu2 mRNA [20]. Further, the distribution of MU2 in larval somatic tissues appeared to be uniform at low magnification over the imaginal discs (Figure S1). In salivary glands MU2 was concentrated on polytene chromosomes with a distribution similar to that of the DAPI stain (Figure 1C).

To study the function of the unmodified MU2 protein, we generated antibodies to an N-terminal fragment of MU2. Antibody staining of Oregon R ovaries confirmed that MU2 was concentrated in the oocyte nucleus at several developmental stages (Figure 1D). A ring shaped localization of MU2 protein was also observed in the cytoplasm of more mature developing egg chambers (Figure S2). In contrast, *mu2^a^* mutants showed low levels of MU2 in their ovaries and poor nuclear localization (Figure 1E). Analysis of the mRNA levels of mu2 using RT PCR showed no significant difference between the wild type and in the mutants, suggesting a lack of nonsense mediated decay. Western analysis of larval and the adult tissues, however, showed a decrease in levels of MU2 protein. Further, the mutants showed a single band for MU2, rather than the two bands seen in wild type (Figure 1F). The presence of a single band with a decreased intensity is consistent with the low signal that we see in immunofluorescence studies. Sequencing of the cDNA from the *mu2^a^* mutant showed that the C-terminal BRCT domain of the protein is obliterated due to a frameshift mutation at amino acid 1065 that leads to a premature stop codon. It is possible that the defective protein is rapidly cleared.

**Figure 2:**
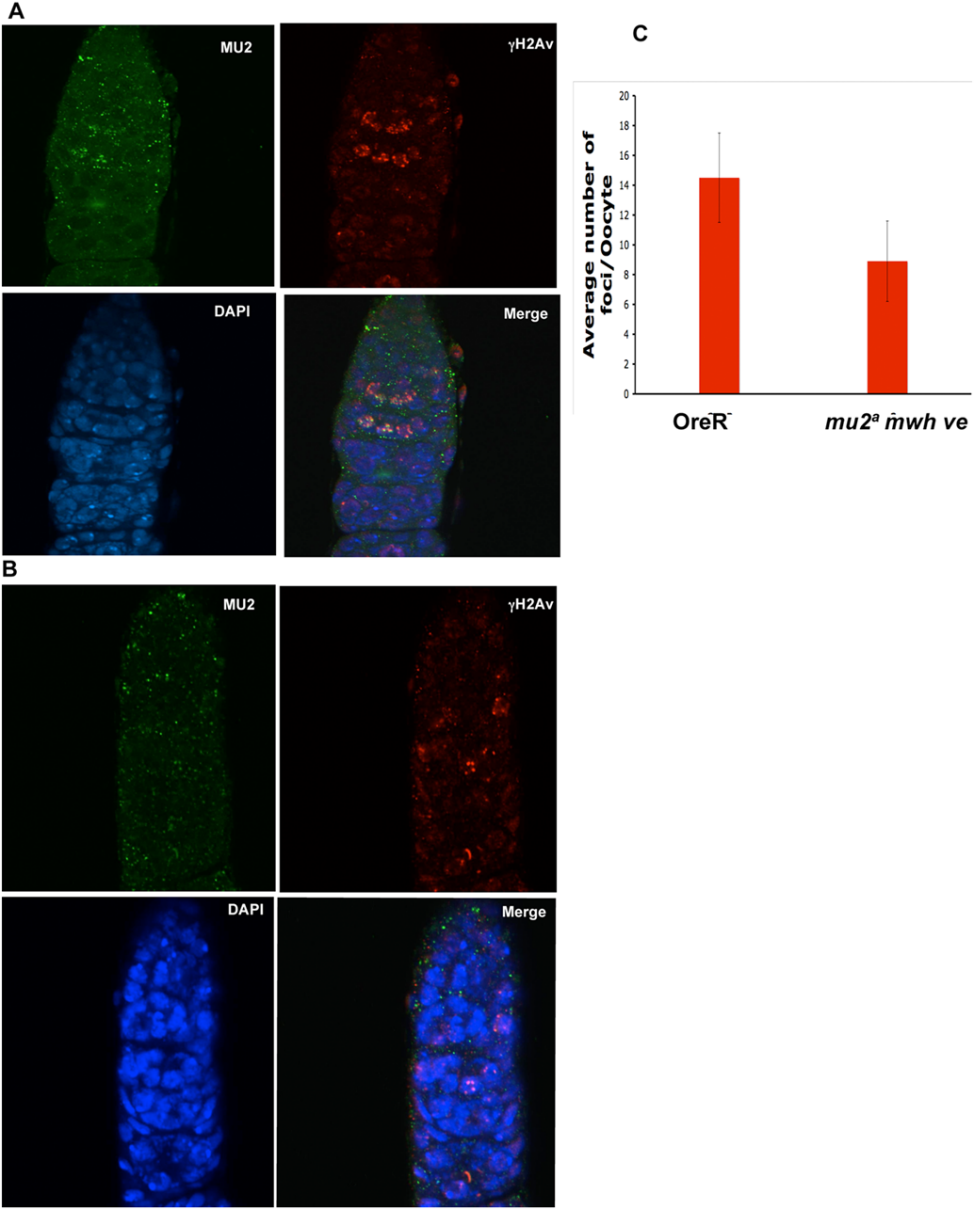
MU2 marks double strand breaks in the germarium. Immunostaining of Oregon R (A) and *mu2^a^* (B) germaria shows γH2Av in red as a mark for DSBs, MU2 in green, and DNA as identified by DAPI in blue. The anterior tip of the germarium is pointing up. MU2 foci co-localize with the γH2Av foci, as shown in the merged image of Oregon R. The small amount of MU2 signal in the *mu2^a^* mutant does not co-localize with γH2Av. (C) Plot showing the difference in the number of γH2Av foci in the germaria of Oregon R and *mu2^a^* ovaries. γH2Av foci were counted in at least ten germaria showing equal intensity of staining without changing any settings of the instruments. The number of foci is represented as the mean±SD.

In *Drosophila* females meiosis occurs within a 16 cell cyst in the germarium at the anterior end of the ovary. The cyst initially contains two pro-oocytes and 14 nurse cells. The two pro-oocytes enter meiosis simultaneously and generate DSBs, although only the actual oocyte nucleus enters the pachytene stage. The other pro-oocyte becomes a nurse cell [22],[23]. We used the antibody to MU2 to examine localization in the germarium. Most of the staining for MU2 was in regions 2A and 2B, which stain specifically with γH2Av antibody. MU2 co-localized with this DSB marker (Figure 2A). However, the germaria of *mu2^a^* females showed very little to no staining with MU2 antibody (Figure 2B). *mu2^a^* germaria also showed a decrease in the number and the intensity of γH2Av foci when compared to wild type (Figure S3), suggesting that the presence of MU2 affects the formation or stability of the γH2Av foci. The decrease in the number of γH2Av foci (Figure 2C) is similar to the decrease in the meiotic recombination seen in the mutant [18]. In both control and mutant oocytes the DSBs were resolved by region 3, as shown by the lack of staining by γH2Av antibody (Figure 2A).

### MU2 Forms Foci in S2 Cells

**Figure 3:**
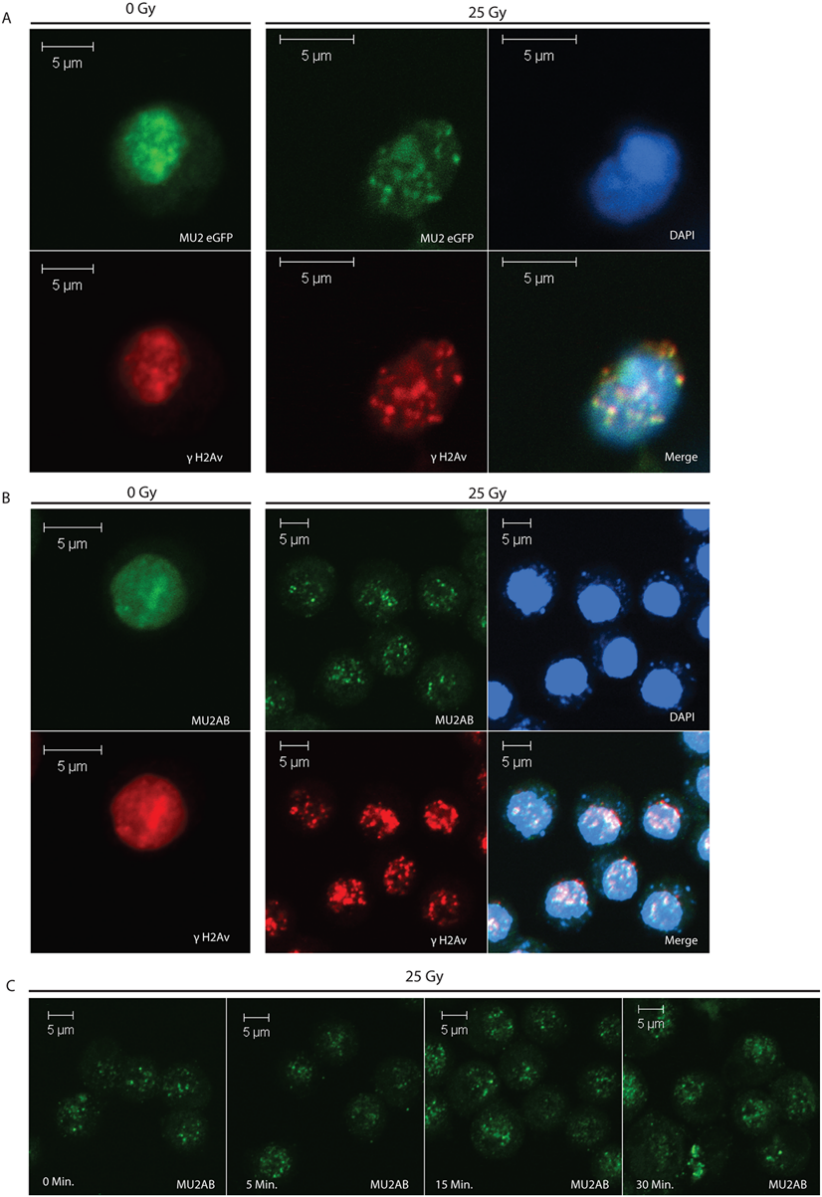
MU2 forms IRIF in S2 cells. (A) S2 cells were transiently transfected with pAGW-MU2 (N-terminal eGFP tag), irradiated with 25 Gy at 5 Gy/min 24 hrs after transfection, and fixed immediately at the termination of treatment. Control S2 cells on the left expressing eGFP-MU2 (green) and stained with γH2Av (red). After irradiation, shown in the middle and right panels, MU2 localized to distinct foci, which co-localized with γH2Av as observed in the merged image (yellow). (B) S2 cells were irradiated with 25 Gy, and stained with anti-MU2 (green) and rabbit anti-γH2Av (red) antibodies. Unirradiated S2 cells are shown on the left; irradiated S2 cells are shown in the middle and right panels. After irradiation, MU2 protein localized to distinct foci and co-localized with γH2Av. (C) Kinetics of the formation of MU2 foci were studied at different intervals after irradiation. MU2 foci appear immediately after irradiation and co-localize with γH2Av.

One of the earliest known responses to DSB formation is the phosphorylation of the C- terminal tail of the variant histone H2A located in the vicinity of the DNA breaks [24]. Antisera to mammalian phosphorylated H2AX (γH2AX) recognize *Drosophila* γH2Av, suggesting a strong homology [21]. γH2AX binds proteins such as MDC1 (through the latter's C-terminal BRCT domain) and acts as a platform for the assembly of DDRs into repair foci. MU2 has a C-terminal tandem BRCT domain, which might interact with γH2Av. We tested this prediction initially by transfecting S2 cells with eGFP-tagged MU2. Unirradiated cells showed a broad distribution of MU2 protein over the nucleus and very little γH2Av (Figure 3A). However, after irradiation, MU2 re-localized to ionizing radiation induced foci (IRIF) and co-localized with γH2Av (Figure 3A).

Similar results were obtained with anti-MU2 antibodies. Staining S2 cells with an anti-MU2 antibody showed that MU2 was distributed broadly in the absence of radiation (Figure 3B). After irradiation, distinct foci of MU2 were observed that were nuclear in nature and co-localized with γH2Av foci (Figure 3B). We further studied the status of the foci at different times after irradiation. MU2 foci appear immediately after irradiation concomitantly with the appearance γH2Av foci (Figure 3C), suggesting that MU2 is intricately involved with recognition of DSBs and the formation of repair foci.

**Figure 4:**
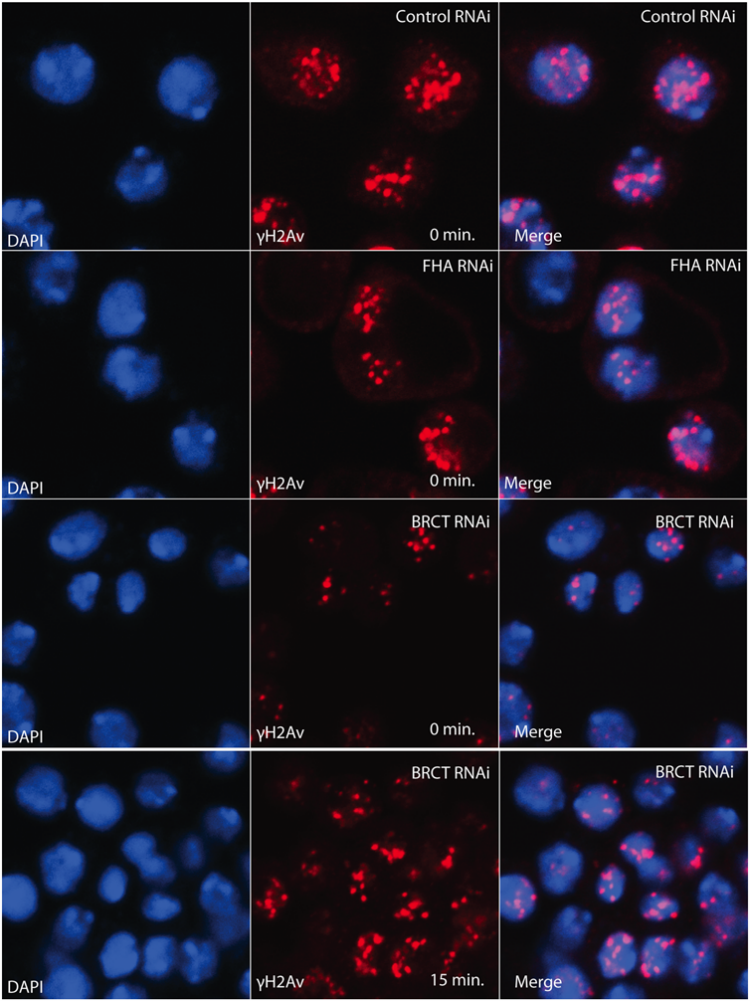
Gamma H2Av foci in S2 cells are depleted by mu2 dsRNA. RNAi was performed in S2 cells targeting two different regions of MU2. After four days of continuous treatment cells were irradiated with 25 Gy at 5 Gy/min, fixed immediately, and stained with rabbit anti-γH2Av (red) antibodies and DAPI (blue). dsRNA treatment with the FHA or the BTCT domain of MU2 are represented as FHA RNAi and BRCT RNAi, respectively, along with a control unrelated RNAi as described earlier. The lower set of panels show γH2Av foci after a chase of 15 min.

To further understand the role of MU2 in the formation of γH2Av foci, we treated S2 cells with dsRNA, targeting two different regions of mu2. RT PCR analysis after 4 days of continuous treatment showed a decrease in the levels of mu2 RNA. S2 cells treated with either N-terminal or C-terminal dsRNA were used to study the effects on γH2Av foci formation. Cells were irradiated, fixed at two different intervals and stained with rabbit anti-γH2Av antibodies to detect γH2Av foci. As shown in Figure 4, a decrease in the levels of mu2 caused a decrease in the number of γH2Av foci immediately after irradiation. However, fixing the cells 15 minutes after irradiation showed an increase in the number and the intensity of foci, although these parameters did not reach wild type levels. These results suggest that MU2 affects the kinetics of foci formation.

### Physical Interaction of MU2 and Phosphorylated H2Av

**Figure 5:**
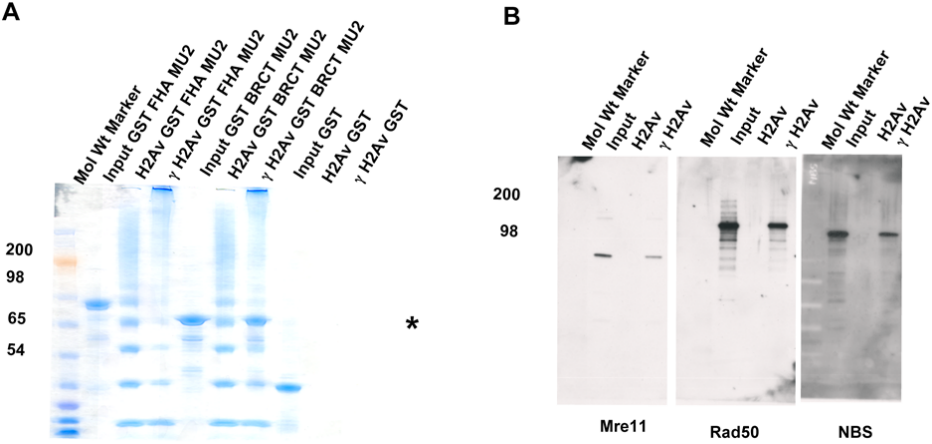
Physical interaction of MU2 and H2Av. Peptide pull-down assays were performed using chemically synthesized peptides of H2Av (phosphorylated and unphosporylated) to examine the interaction between bacterially expressed domains of MU2 and the peptides of H2Av. The H2Av peptides were biotinylated at the N terminus, which was used to conjugate them to the streptavidin agarose beads. These beads were then incubated with bacterially expressed and purified GST, GST-FHA, GST-BRCT domains of MU2. Panel (A) shows a coomassie-stained SDS polyacrylamide gel of an H2Av peptide pull-down experiment (γH2Av indicates the phosphopeptide; H2Av indicates the unphosphorylated peptide). Expressed GST-FHA and GST-BRCT domains, and bands appearing at the same molecular weight in other lanes, were identified using mass spectrometry. The GST-BRCT domain is pulled down specifically by the phosphorylated form of the peptide (position of the band indicated by an asterisk). (B) Biotinylated H2Av (phosporylated or unphosphorylated) peptides were incubated with nuclear extracts from S2 cells to isolate the MRN complex. Ten mg of total protein from the nuclear extract was loaded onto agarose beads. At the end of incubation the beads were washed, and the MRN complex was detected by Western analysis.

To further understand the nature of the interaction between MU2 and γH2Av, we performed a peptide pull-down assay using the C-terminal amino acids of H2Av phosphorylated at the conserved serine at position 137 as a probe for the bacterially expressed N- (aa 1–250) and C-terminal (aa 1019–1259, BRCT) domains of MU2 (Figure 5A). The BRCT domain, indicated by an asterisk, interacted specifically with the phosphorylated H2Av peptide. The N terminus of MU2, however, did not show any interaction with either the phosphorylated or the unphosphorylated peptides. The bands that are observed in the lanes of unphosphorylated peptide at the positions of the GST inputs were confirmed to be of bacterial origin by mass spectrometry, suggesting that the unphosphorylated peptide does not show any binding to the MU2 domains in vitro. The direct physical interaction between the BRCT domain of MU2 with the phospho-peptide suggests that MU2 is an adaptor protein, and that the N terminus might act as a base for the recruitment of other proteins.

Using a peptide pull-down assay, we analyzed the proteins that may form a complex with MU2 in nuclear extracts of S2 cells. Immunoblots were performed on the pull-down lanes using antibodies to the components of the MRN complex. MRE11, RAD50 and NBS were detected in the γH2Av lane of the pull-downs (Figure 5B). These results suggest that while binding to the γH2Av at the C terminus, MU2 also binds a complex of proteins, possibly at the N terminus, and thereby recruits the DDR complex to the site of a DSB.

### The MU2 FHA Domain Interacts with the MRN Complex

Domain searches identified the presence of an FHA domain in the N-terminal region of *D. melanogaster* MU2 (aa 9–106) and five other *Drosophila* species [25],[26]. Proteins containing FHA domains bind phosphorylated serine and threonine residues of proteins involved in DNA damage signaling. Since MU2 is a component of meiotic recombination foci and radiation induced foci, we set out to identify the domain of MU2 that interacts with MRN complex.

**Figure 6:**
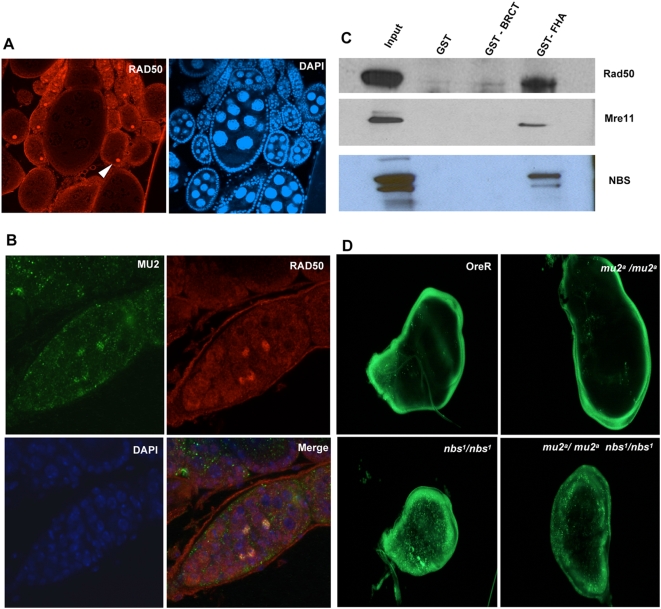
Interaction of MU2 with MRN complex. (A) Immunostaining of an Oregon R ovary with rabbit anti-RAD50 detected with secondary goat anti-rabbit IgG AlexaFluor 594 (red), and counterstained with DAPI (blue). (B) Germarium of an Oregon R ovary stained with rabbit anti-RAD50 (red), mouse anti-MU2 (green), and DAPI (blue) showing co-localization of RAD50 and MU2 in foci (yellow). (C) Immunoblot analysis of the eluates of the GST pull-down experiments detecting the physical interaction between the N-terminal FHA domain of MU2 and components of the MRN complex. (D) Genetic interaction between *nbs* and *mu2* was assessed by studying the levels of apoptosis in Oregon R, homozygous *nbs* and *mu2^a^* mutants, and *mu2^a^ nbs* double mutants. Wing imaginal discs were dissected, and propidium iodide staining was performed to detect apoptosis.

In the vitellarium of the *Drosophila* ovary, RAD50 is found in somatic follicle cells and oocyte nuclei (Figure 6A). Staining of the germarium with anti-RAD50 antibody showed that MU2 and RAD50 colocalize at the sites of recombination foci (Figure 6B). These results suggest the presence of a multiprotein complex, including γH2Av, MU2 and the MRN complex, that is involved in the recognition and repair of a DSB. To identify the domain of MU2 that interacts with the MRN complex, we created GST fragments of the N-terminal FHA and the C-terminal BRCT domains of MU2 and incubated these fragments with nuclear extracts from S2 cells. Immunoblot analysis of GST pull-downs using antibodies to MRE11, RAD50 or NBS showed that the N-terminal FHA domain of MU2 interacts with the MRN complex, confirming the presence of a functional FHA domain at the N terminus (Figure 6C).

We next asked whether there could be a genetic interaction between MU2 and the components of the MRN complex. Mutations in the genes of the MRN complex are homozygous pupal lethal, and the wing imaginal discs of these homozygous animals show high levels of spontaneous apoptosis. Mutations in certain components of the DDR pathway are known to decrease the levels of spontaneous apoptosis. Thus, we predicted that, if MU2 is a transducer of the signal to repair a spontaneous or an induced DSB, *mu2^a^* should reduce the number of apoptotic cells. We examined apoptosis in wild type, homozygous single and double mutants of *nbs^1^* and *mu2^a^*. *mu2^a^* mutants do not show any increase in apoptosis compared with the controls, whereas the *nbs^1^* mutants show high levels of spontaneous apoptosis (Figure 6D). Interestingly, there is a considerable decrease in the levels of spontaneous apoptosis in the wing discs of the *mu2^a^ nbs^1^* double mutants (Figure 6D). However, *mu2^a^* did not significantly decrease the levels of apoptosis in the *rad50* and the *mre11* mutants. These results suggest a possible role for NBS separate from the MRN complex in apoptotic signaling and repair, as has been suggested previously [27],[28].

### MU2 Is Homologous to Human MDC1

**Figure 7:**
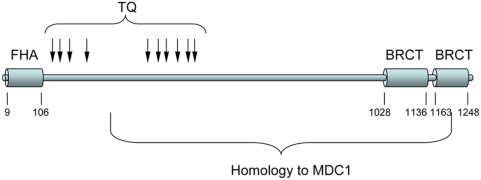
Structure of MU2. Diagram of the MU2 protein showing the N-terminal FHA domain and the C-terminal tandem BRCT domain. Arrows show the positions of threonine-glutamine (TQ) dipeptides. Numbers indicate the amino acid positions. Homology to MDC1 is based on a blast2 comparison using BLOSUM45.

If MU2 plays a conserved role in the formation of IRIFs, homologs in other organisms should be evident. A blast search of insects outside of diptera shows only one strong hit (E = 6.8e–9), which is to a protein ‘*similar to mediator of DNA damage checkpoint 1*’ (MDC1) in the wasp, *Nasonia*. Similarity extends along the carboxy half of the *Drosophila* MU2 protein from aa 581 to 1251. A blast of the human genome reveals only one hit, to MDC1 (E = 6e–6). The similarity, however, is restricted to the BRCT domain. Alignments of *Drosophila* MU2 isoform PB and human MDC1 using blast2 indicate more extensive homology to MDC1 (aa 289–1203 of MU2). Thus, the only region that does not show similarity between *Drosophila* MU2 and human MDC1 is the N terminus, which carries an FHA domain in both proteins. As in MDC1, MU2 contains several threonine-glutamine (TQ) dipeptides in the N-terminal half of the protein (Figure 7).

### MU2 Is Important for the Intra-S Checkpoint

**Figure 8:**
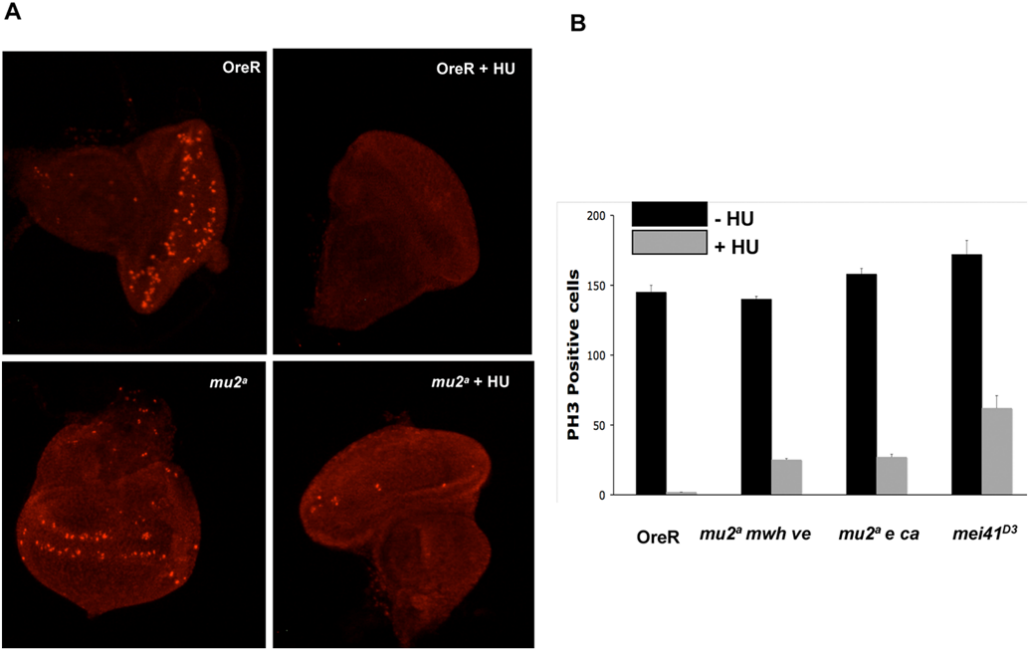
MU2 is involved in the intra S-phase checkpoint. (A) Eye imaginal discs from Oregon R and *mu2^a^* larvae were treated with 0.05 M HU for 2.5 h. Mitotic cells were assessed by staining the discs with the mitosis-specific marker phospho-histone 3 (PH3) Ser 10 followed by fluorescence microscopy. (B) Plot showing the number of mitotic cells in each genotype in the presence and absence of HU. Five discs were assayed for the number of mitotic cells. The *mu2^a^* mutant was assayed in two different genetic backgrounds.

If MU2 is a homolog of MDC1, it is possible that it modulates the intra-S checkpoint [29]. As eye imaginal discs develop, a morphogenetic furrow passes over the disc from posterior to anterior. In this furrow cells undergo a final wave of synchronous mitoses. Thus, the third instar eye disc displays a row of mitotic cells. To test whether a mutation in *mu2* has an effect on cell cycle control, eye discs were treated with 0.05 M hydroxyurea (HU) for 2.5 h, and mitotic cells were detected by antibodies to histone H3 phosphorylated on Ser 10 (PH3). As the eye-antennal imaginal discs in *mu2^a^* larvae were larger than those in Oregon R, an effort was made to score discs of equal size. The HU treated control discs did not show a discernible mitotic wave (Figure 8A) and had very few PH3 spots. However, there was a 10 fold increase in the number of cells undergoing mitosis in the *mu2^a^* discs after HU treatment compared with Oregon R (Figure 8B). As a positive control, we tested *mei41^D3^* mutant discs for progression to mitosis after treatment with HU. These discs exhibited a 3 fold increase in mitotic cells over *mu2^a^* and a 30 fold increase over Oregon R (Figure 8B). Thus, our studies of the intra-S checkpoint using eye-antennal imaginal discs indicate that MU2 plays a role in the control of this process.

## Discussion

Although rare, terminal deficiency chromosomes that have lost a native telomere and gained a neotelomere have been demonstrated in humans [30], plants [31], ciliates [14], yeast [32], and plasmodium [33]. One classic example is the *Drosophila mu2^a^* mutation that enhances the recovery of terminal deficiencies from irradiated oocytes [12]. Genetic studies suggested that *mu2^a^* mutants reduce the rate of DNA repair during oogenesis, that the unrepaired broken chromosomes bypass cell cycle control, and that the broken ends are healed by the addition of new telomeres after fertilization [17]. Differences in the repair capacity of lesions induced in sperm and oocyte pronuclei after fertilization suggested that MU2 might be chromosomal or at least restricted to the oocyte nucleus. Chromatin structure is known to play a key role in the repair of DSBs, such that the sites of DSB are devoid of conventional nucleosomes [34] and many key chromatin components, such as HP1 [35]. Our studies show that the MU2 protein is nuclear and at least in salivary cells localizes to chromosomes, confirming its association with chromatin. Further, the presence of MU2 is important for the formation and/or the stability of IRIFs, which in turn are necessary for alterations in chromatin structure that are prerequisite for repair.

### MU2 Is an Ortholog of Human MDC1

We propose an explanation for the *mu2^a^* mutant phenotype by suggesting that *mu2* is an ortholog of human MDC1. MDC1 forms a major component of IRIFs in mammalian cells by binding γH2AX using its C-terminal BRCT domain [36] and the MRN complex using its N-terminal FHA domain. Thus, MDC1 acts as a scaffold for the assembly of the IRIF, the multiprotein complex in which DSB repair occurs. MEFs isolated from MDC1 null mice do form γH2AX foci, but the kinetics of focus formation are affected. We used RNAi of MU2 in S2 cells to understand the role of MU2 in the formation of γH2Av foci. Our studies indicated that foci are formed after irradiation, but their number and intensity are reduced. Further, the kinetics of formation are affected. These results are in agreement with a decrease observed in the germarium of the ovaries. MDC1 plays an important role in the regulation of the intra-S phase [37] and G2/M checkpoints [38] by regulating the corresponding effecter proteins. It also enhances NHEJ at dysfunctional telomeres [39], showing that proteins involved in recognition of DNA damage are important in telomere dynamics. MDC1 knockout mice are viable, although male MDC1^−/−^ mice are sterile, suggesting a defect in spermatogenesis or meiosis [40]. MDC1 knockout mice and the cell cultures established from them, however, are hypersensitive to ionizing radiation. The phenotypes of the MDC1^−/−^ mice were very similar to those observed for H2AX^−/−^ mice [40].

BLAST searches show good homology between *Drosophila* MU2 and human MDC1, and domain searches indicate that MU2 contains an N-terminal FHA domain, a C-terminal BRCT domain, and a region of concentrated TQ dipeptides, as found in MDC1. We have shown that the C-terminal BRCT domain of MU2 interacts with γH2Av, while the N-terminal FHA domain binds the MRN complex. MU2 co-localizes with γH2Av at repair foci in S2 cells and at recombination foci during meiosis. *mu2^a^* mutants are viable, and exhibit defects in meiotic recombination [18] and in the intra-S checkpoint, although they are not hypersensitive to killing by ionizing radiation or radiomimetic chemicals [17]. The lack of radiation sensitivity seen in *mu2* mutants may be because we have not found a null allele. Thus, by these measures MU2 appears to have the same structure and perform the same functions as MDC1.

### MU2 and the Generation of New Telomeres

The notion that MU2 is an ortholog of MDC1 suggests that MU2 is necessary for an early step in lesion recognition and IRIF formation. Thus, DNA breaks induced in *mu2* mutant oocytes would not be recognized properly. As a result, they would not induce cell cycle delay and would not be amenable to repair. Unrepaired broken chromosomes introduced into the zygote after completion of meiosis would then be subject to healing, i. e. the acquisition of a new telomere. In contrast, when males are irradiated and crossed to *mu2^a^* females, no terminal deficiencies were found, while fertility remained high. Thus, lesions in sperm seem to be repaired when placed in a *mu2^a^* mutant cytoplasm, and the effects of MU2 are limited to the oocyte nucleus. These chromosome breaks in the sperm pronuclei are generally repaired before syngamy, as evidenced by a paucity of mosaics among the mutants that are found [41]. If recognition of the damage by MU2 were necessary, then the repair of damage in the sperm should also be compromised in the absence of the MU2 protein, which however is not the case. What is the difference between radiation induced lesions in oocyte chromosomes and in sperm chromosomes? There are at least two possibilities.

One possibility is that sperm chromosomes are packaged using arginine-rich histones. Chromatin remodeling and eviction of nucleosomes is required for IRIF formation in standard chromatin. Such eviction of conventional nucleosomes may not be required in the case of sperm chromatin, as it is already devoid of the lysine-rich nucleosomes, and the arginine-rich histones must be stripped away to allow chromosome decondensation in preparation for replication and syngamy. This may allow access of the induced lesion to repair enzymes in the absence of IRIFs.

**Figure 9:**
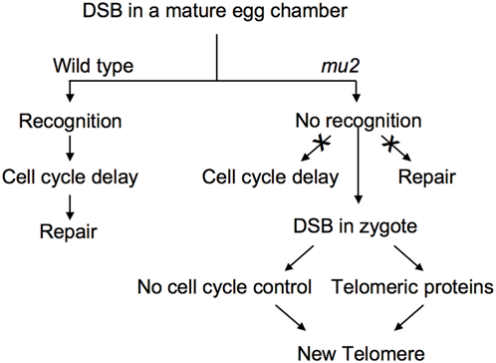
Model to explain new telomere formation in *mu2* mutants. In a wild type genetic background a DSB induced by irradiation in a stage 14 egg chamber leads to the activation of a DNA damage response checkpoint and DNA repair. In *mu2^a^* mutants recognition of a DSB is impaired. Thus, the damage response checkpoint is not activated and DNA repair does not ensue. This leads to incorporation of the unrepaired break into the zygote. The lack of cell cycle control in the early embryo (until cycle 10) and the presence of relatively high levels of telomeric proteins (deposited maternally) may lead to the association of the DSB with these proteins and the establishment a new telomere.

Alternatively, a DSB introduced into a sperm chromosome would be amenable to repair because the break produces two broken chromosome ends that would be maintained in the same nucleus and thus available for NHEJ or HR to restore the chromosome. A broken chromosome in a mature oocyte, on the other hand, would only put a single broken chromosome end into the zygote after the completion of the meiotic divisions, because the acentric fragment would not move to the pole at anaphase and would be lost. Thus, this single broken chromosome end in the oocyte pronucleus would not be amenable for either NHEJ or HR. As there is no cell cycle control in the early embryo [42], the broken chromosome ends may have a chance to associate with the telomeric proteins HP1 and HOAP, which are present in abundance in the embryo, and become established as new telomeres (Figure 9). After syngamy the new diploid nucleus undergoes a rapid series of divisions to produce a thousand nuclei with 16,000 telomeres within 1–2 hours. Thus, the young embryo seems poised to generate a large number of new telomeric ends in a very short time. This may explain why an unrepaired chromosome break may acquire a new telomere at a high rated during development of the young embryo. This result is reminiscent of work in maize, where broken chromosome ends are healed specifically upon entry into the zygote [10]. Our observations provide a biochemical and a molecular basis for this phenotype.

## Materials and Methods

### *Drosophila* Strains, Plasmids, and Constructs

*Drosophila* stocks were maintained at 25° C on corn meal, molasses medium with dry yeast added to the surface. Oregon R was used as a control for all experiments. Full length mu2 mRNA was amplified from total RNA using RT-PCR and cloned into a pAGW destination vector. The C2 genomic fragment of *mu2* that rescues the mutant phenotype [20] was cloned into pCaSpeR-DEST2 obtained from the *Drosophila* Genomics Resource Centre. The N-terminal (aa 1–250) and C-terminal domains (aa 1019–1259) of MU2-PB were cloned in frame at the EcoRI site into pGEX-4T1 to express them as GST fusion fragments. The expressed proteins were detected using mass spectrometry and were immobilized on glutathione sepharose (Amersham) for further studies. pCaSpeR-DEST2 was injected into *y w^1118^* embryos, transformants were recovered, and the insertions were mapped genetically.

### Production of Anti-MU2 Antibodies

A 5′ fragment of *mu2* (bp 37–678) was amplified from cDNA using primers containing the EcoRI restriction sites and cloned into pGEX4T1. The purified fragment was identified using mass spectrometry and was used to generate polyclonal antibodies in mice.

### Irradiation and Immunostaining of S2 Cells

Exponentially growing S2 cells were seeded in 8 well chambered slides and transfected with pAGW-dMU2 plasmid using Effectene (Qiagen). Cells were exposed to 25 Gy of γ irradiation using ^137^Cs at 5 Gy/min. Immunostaining of S2 cells was performed as described [43]. Cells were incubated with primary rabbit anti-γH2Av at 1∶1000 dilution (Rockland Biochemicals, MD) and with mouse anti-MU2 antibodies (1∶100). Slides were mounted in SlowFade Gold antifade reagent (Invitrogen) and were visualized using confocal microscopy.

### MU2 RNAi in S2 Cells

RNAi was performed in S2 cells according to established protocols [44],[45]. Two regions of the mu2 gene, the N-terminal FHA domain, and the C-terminal tandem BRCT domains, were amplified from mu2 cDNA using primers with T7 promoter binding sites at the 5′ and the 3′ ends. As a negative control, we PCR amplified a 750 bp sequence from the bacterial cloning plasmid using the strategy described above. The PCR product was gel purified, an in vitro transcription reaction was performed, and the dsRNA was purified using Megascript T7 kit (Ambion Inc.) according to the manufacturers instructions. S2 cells were grown as described previously, plated at a density of 1×10^5^ and transfected with 1 µg dsRNA using Effectene (Qiagen) according to manufacturers instructions. Cells were exposed to 25 Gy of γ irradiation using ^137^Cs at 5 Gy/min and immunostaining was performed as described in the previous section.

### Ovary Fixation and Immunofluorescence

Fixation and staining of the ovaries was performed according to Scott and Hawley [46]. Ovaries were stained with mouse anti-MU2 serum (1∶100), mouse anti-C(3)G antibodies (1∶200), rabbit anti-Rad50 antibodies (1∶200), or rabbit anti-γH2Av (1∶1,000). The γH2Av foci were counted manually in the germaria that showed clear foci.

### Preparation of Nuclear Extracts

Nuclear extracts were prepared from S2 cells using the protocol of Dingham et al. [47]. Protein concentrations were estimated and extracts were stored at −70°C.

### GST Pull-Down Assays

A GST pull-down assay was performed to identify the region of MU2 that interacts with the MRN complex. Nuclear extracts were prepared from S2 cells as described above, were used as a bait and incubated with GST, GST-FHA, GST-BRCT fragments of MU2 that act as prey to capture the MRN group of proteins in the nuclear extracts. The GST preys were loaded onto the glutathione agarose beads and incubated with nuclear extracts prepared from S2 cells overnight in NETN (20 mM TrisHCl pH 8.0, 0.5 mM EDTA, 0.1% NP40, 100 mM NaCl) buffer containing 2% BSA. The beads were washed extensively to separate the unbound from the bound proteins, boiled in SDS sample buffer and analyzed by western blotting for the detection of proteins of MRN complex.

### Peptide Binding

Synthetic peptides corresponding to the C-terminal tail of H2Av (N-KEETVQDPQRKGNVIL**S**QAY-C) phosphorylated at the Ser residue were generated chemically, along with unphosphorylated controls, and biotinylated at the N terminus (Sigma Genosys). The peptides were conjugated to streptavidin agarose beads. Pull-downs were performed using the nuclear extracts from S2 cells (Figure 5B) or the GST, GST-FHA, and GST-BRCT domains of MU2 expressed in bacteria (Figure 5A) acccording to Stucki et al. [36]. Bound proteins were subjected to immunoblotting to detect complexes from nuclear extracts and to SDS PAGE for the GST fragment pull-downs.

### Apoptosis and Determination of S-Phase Checkpoint

Wing imaginal discs were dissected and stained with a the vital dye acridine orange as described previously [48],[49]. Determination of S phase checkpoint was performed according to Smolik and Jones [50] using Oregon R and *mu2^a^* flies. The total number of mitotic cells was counted in at least five discs. Each experiment was performed three times.

### Database Searches

Domain searches were conducted at four sites: NCBI (http://www.ncbi.nlm.nih.gov/Structure/cdd/cdd.shtml), Prosite (http://ca.expasy.org/prosite/), Superfamily (http://supfam.cs.bris.ac.uk/SUPERFAMILY/), and Pfam (http://pfam.sanger.ac.uk/search). Blast searches against *Drosophila* and other insect species as performed at FlyBase (http://flybase.bio.indiana.edu/blast/). Blast searches against the human genome was performed at NCBI (http://www.ncbi.nlm.nih.gov/blast/Blast.cgi). As comparisons among the 12 *Drosophila* genomes indicated that the MU2 amino acid sequence had diverged rapidly, blast searches were done using BLOSUM45 and filters turned off. The cutoff E value was set at 0.001.

## Supporting Information

Figure S1 Distribution of MU2 in the imaginal discs. Imaginal discs were dissected from wandering third instar larvae. The discs were fixed in 4% paraformaldehyde and immunostained with mouse anti-MU2 antiserum. (A) Eye antennal imaginal disc (B) wing imaginal disc and (C) leg imaginal disc. MU2 is distributed evenly over the discs.
(47.2 MB TIF)

Figure S2 Distribution of MU2 in the oocyte cytoplasm. Immmunostaining of Oregon R ovaries using mouse anti-MU2 antiserum. Concentration of the fluorescent signal is clearly visible in the nucleus (arrow) and a ring shaped pattern is observed at the anterior end of the cytoplasm of the oocyte (arrowhead).
(4.21 MB TIF)

Figure S3 Formation of recombination foci during meiosis. Immunostaining of germaria from ovaries of Oregon R (A) and *mu2^a^* (B) shows γH2Av (red) to detect DSBs, and C(3)G (green) to detect the synaptonemal complex (SC). The merged image shows that most of the DSBs localized to the SC. γH2Av foci were detected mostly in region 2A, with some in region 2B, but were mostly absent from the region 3.
(1.56 MB TIF)
