# Supplementary figures and images for: Recognition of Double Strand Breaks by a Mutator Protein (MU2) in Drosophila melanogaster
Source: PLoS Genet. 2009 May 8;5(5):e1000473. doi: 10.1371/journal.pgen.1000473 (PMC2672170; doi:10.1371/journal.pgen.1000473)

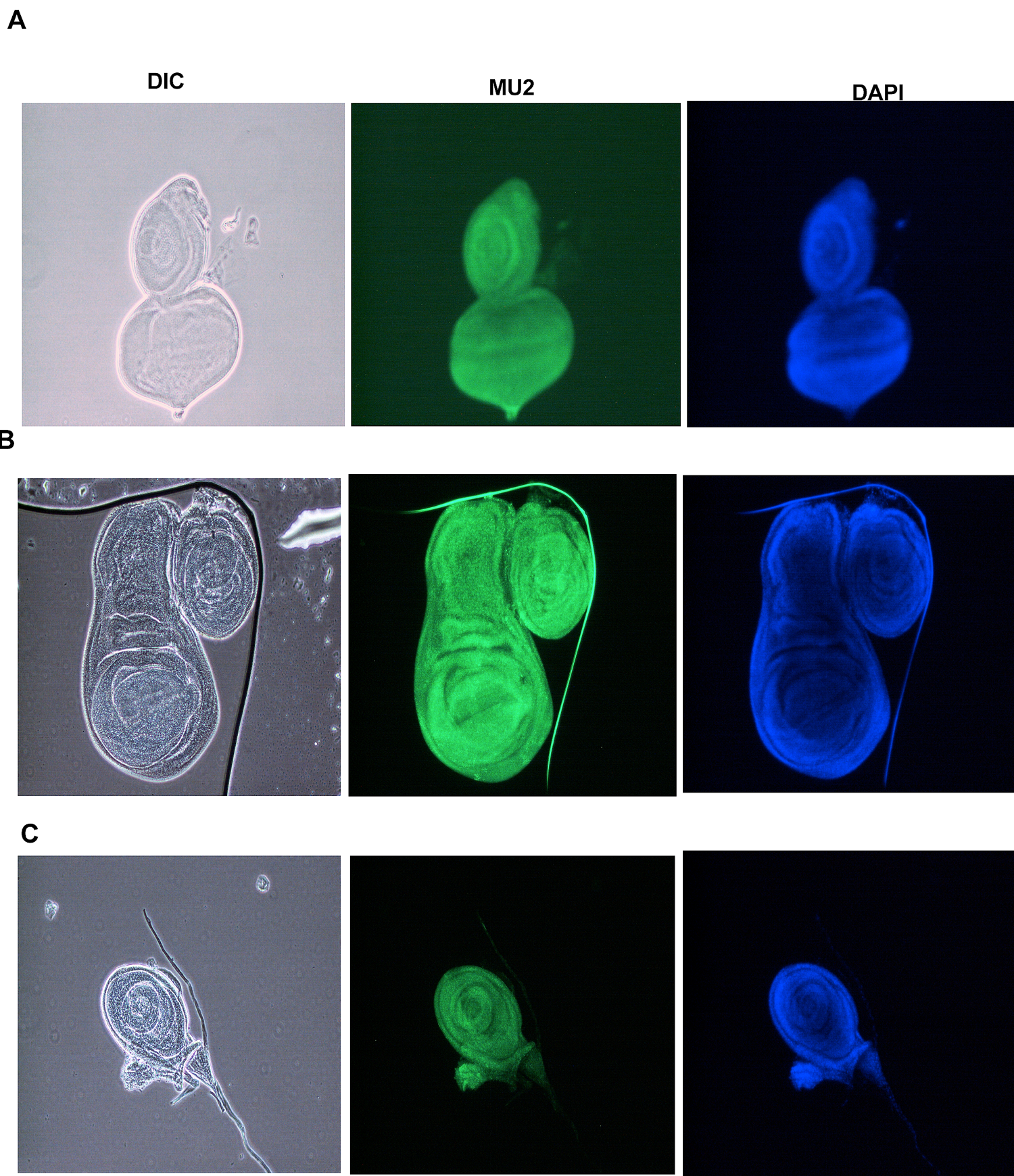

Supplement: Figure S1 — Distribution of MU2 in the imaginal discs. Imaginal discs were dissected from wandering third instar larvae. The discs were fixed in 4% paraformaldehyde and immunostained with mouse anti-MU2 antiserum. (A) Eye antennal imaginal disc (B) wing imaginal disc and (C) leg imaginal disc. MU2 is distributed evenly over the discs. (47.2 MB TIF) [file pgen.1000473.s001.tif]

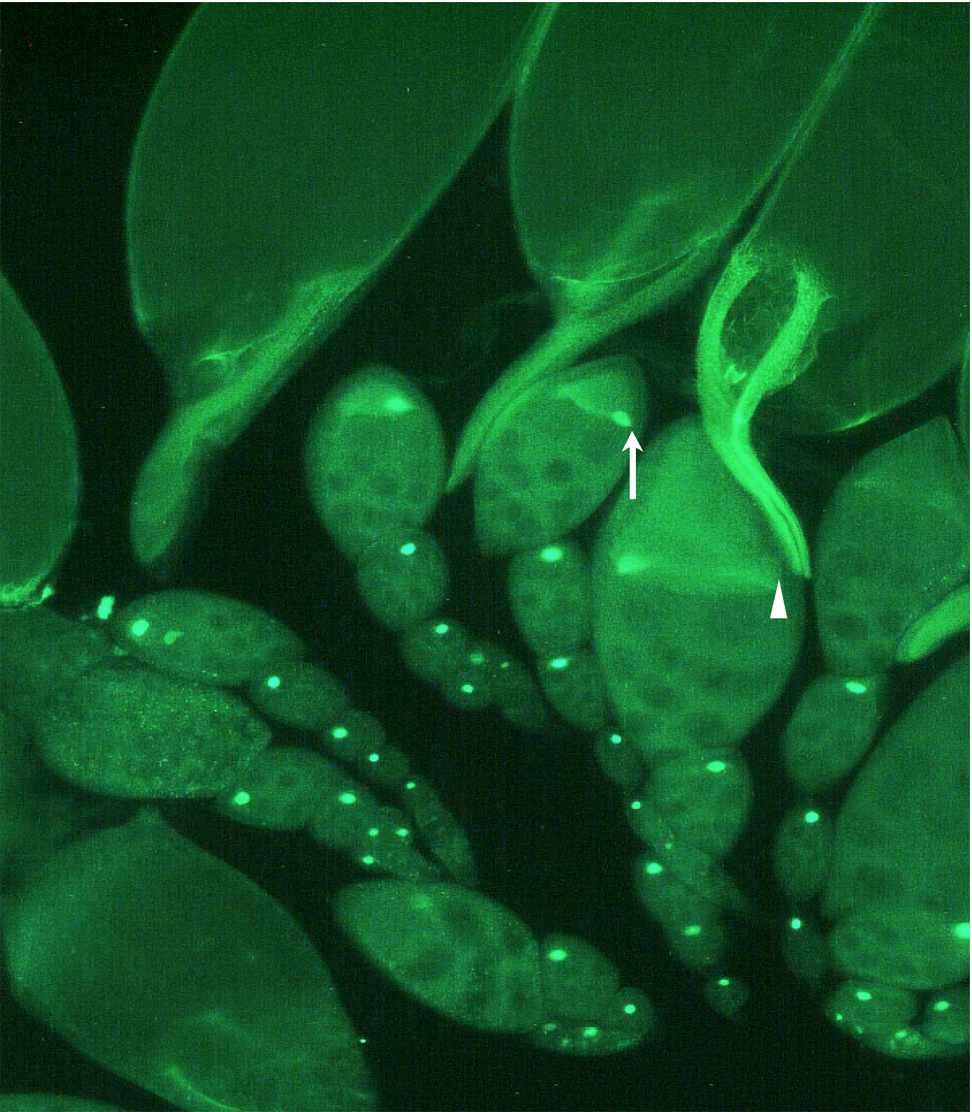

Supplement: Figure S2 — Distribution of MU2 in the oocyte cytoplasm. Immmunostaining of Oregon R ovaries using mouse anti-MU2 antiserum. Concentration of the fluorescent signal is clearly visible in the nucleus (arrow) and a ring shaped pattern is observed at the anterior end of the cytoplasm of the oocyte (arrowhead). (4.21 MB TIF) [file pgen.1000473.s002.tif]

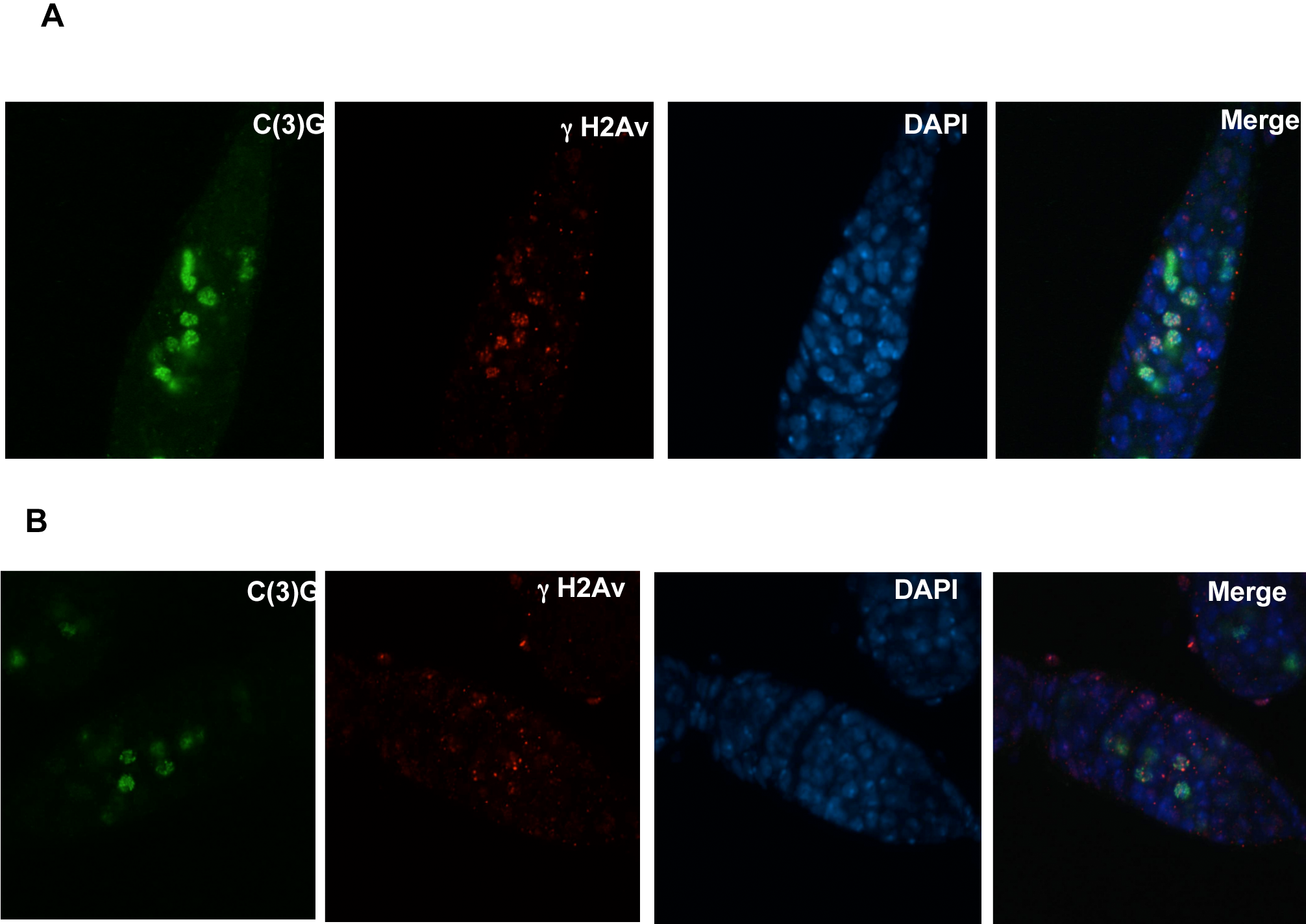

Supplement: Figure S3 — Formation of recombination foci during meiosis. Immunostaining of germaria from ovaries of Oregon R (A) and mu2a (B) shows γH2Av (red) to detect DSBs, and C(3)G (green) to detect the synaptonemal complex (SC). The merged image shows that most of the DSBs localized to the SC. γH2Av foci were detected mostly in region 2A, with some in region 2B, but were mostly absent from the region 3. (1.56 MB TIF) [file pgen.1000473.s003.tif]
